# Efficacy and safety of immunotherapy in head and neck tumors: a systematic review and meta-analysis of clinical outcomes and survival benefits

**DOI:** 10.3389/fonc.2026.1845660

**Published:** 2026-05-15

**Authors:** Chuang Qi, Xin Zhang, Hao Guo

**Affiliations:** Department of Oncology, The Central Hospital of Enshi Tujia and Miao Autonomous Prefecture, Enshi, Hubei, China

**Keywords:** efficacy, head and neck squamous cell carcinoma, immune checkpoint inhibitors, immunotherapy, meta-analysis, PD-1, PD-L1, real-world evidence

## Abstract

**Background:**

The treatment of head and neck squamous cell carcinoma (HNSCC) poses significant difficulties because patients with advanced disease experience poor survival rates. The use of immune checkpoint inhibitors as therapy shows potential, yet researchers report inconsistent findings regarding their effectiveness and safety in clinical trials and real-world settings.

**Objective:**

The study aimed to assess the effectiveness and safety of immunotherapy for HNSCC, as well as treatment success by biomarker status, through a systematic review and meta-analysis of clinical and real-world evidence.

**Methodology:**

The researchers conducted a comprehensive systematic review and meta-analysis that included all available randomized controlled trials, phase II and III studies, retrospective cohort studies, and real-world evidence. The researchers applied a random-effects model analysis, using inverse-variance weighting and a Freeman–Tukey double arcsine transformation, to pool the data. The I² statistic served as the measure of heterogeneity, while publication bias was assessed using funnel plots and Egger’s test. The GRADE methodology assessed the certainty of the evidence.

**Results:**

The analysis included 36 studies involving 8880 to 5930 patients, assessed across five main outcome categories. The clinical effectiveness assessment showed a combined rate of 0.16 (95% CI: 0.14–0.18), with considerable heterogeneity (I² = 81%). The effectiveness assessment showed a combined rate of 0.18 (95% CI: 0.16–0.19), which displayed evidence of publication bias. The combination therapies achieved an improvement rate of 0.18 (95% CI: 0.15–0.22), whereas the dual/targeted immunotherapy showed superior performance, with an improvement rate of 0.22 (95% CI: 0.19–0.24), based on highly reliable evidence. The PD-L1-based outcomes showed a pooled proportion of 0.17 (95% CI: 0.16–0.19) with moderate heterogeneity (I² = 66%). The majority of randomized controlled datasets showed no evidence of publication bias.

**Conclusion:**

Immunotherapy delivers moderate yet dependable medical results for HNSCC, with its greatest effectiveness achieved through dual- or targeted-treatment methods. The randomized research evidence shows strong treatment effects, whereas the actual treatment data reveal different results and suggest possible publication bias. The PD-L1 biomarker is a useful tool for predicting response, yet its performance is inconsistent across applications. The use of immunotherapy represents a major development in HNSCC treatment.

## Introduction

Head and neck cancers (HNCs), mainly head and neck squamous cell carcinoma (HNSCC), are a major health burden in the world, with enormous morbidity and mortality rates worldwide. Global cancer estimates indicate that HNCs are one of the most prevalent types of malignancies that present hundreds of thousands of new cases and deaths in a wide range of populations yearly (1, 2). Recent statistics show that the incidence is increasing, especially in low- and middle-income nations, highlighting disparities in health care access and prevention measures (3). HNSCC develops from the mucosal lining of the oral cavity, oropharynx, hypopharynx, and larynx and is characterized by aggressive behavior and complex tumor biology (4, 5). Over the past decades, the epidemiology of HNSCC has changed greatly, in large part because of the rising importance of human papillomavirus (HPV)-related oropharyngeal cancer. The positive HPV tumors have a unique molecular phenotype and a favorable prognosis over HPV negative disease, which has been largely attributed to conventional risk factors, including tobacco and alcohol use (6). Although the prevention and early disease detection have improved, a significant percentage of patients continue to arrive with advanced-stage disease, which has led to poor overall survival rates.

Traditional methods of treatment of HNSCC, such as surgery, radiotherapy, and cytotoxic chemotherapy, have been a part of the mainstay of treatment. Although multimodal approaches have improved locoregional control, survival benefits remain limited, especially in recurrent or metastatic settings (7). Epidermal growth factor receptor (EGFR) and other targeted therapies have shown moderate improvements in outcomes but are often accompanied by resistance and toxicity (8). The therapeutic options that can be used in recurrent or metastatic settings are limited, with high adverse effects and undermining the quality of life. An example is that chemotherapy-based treatment regimens only have moderate survival gains, and overall median survival is less than one year in most instances (9). This restriction makes it even clearer that more efficient and less toxic treatment options are required.

There is growing clinical evidence of the effectiveness of ICIs in HNSCC. Educational randomized trials have shown improved survival rates with agents such as pembrolizumab compared with conventional treatment plans. Preclinical trials also established significant antitumor efficacy and tolerable safety in highly pretreated patients. Moreover, immunotherapy has demonstrated long-lasting responses in biomarker-unselected patients, thereby offering broad clinical applicability. In addition to clinical effectiveness, advances in tumor immunology have deepened our understanding of the tumor microenvironment and the immune response mechanisms in HNSCC. Tumor mutational burden and genomic profiling, which are based on biomarker development, have become significant predictors of immunotherapy response. In addition, molecular predictors, including frameshift mutations and immunogenic gene signatures, have shown potential for identifying patients best suited to receive ICIs. The recent literature also points to a changing therapeutic landscape for HNSCC, as more immunotherapy components are incorporated into the standard treatment paradigm, whether through combinations or sequential therapies (10).

This discrepancy highlights the need for a recent, comprehensive meta-analysis to provide a clearer picture of the clinical value of immunotherapy in head and neck cancers. As such, the current systematic review and meta-analysis are designed to assess the efficacy and safety of immunotherapy in head and neck tumors, with particular attention to survival and adverse events. The study aims to synthesize evidence from randomized trials and real-world studies to provide clinically meaningful information to guide therapeutic decision-making and future research directions in the age of precision oncology.

## Methodology

### Study design and data sources

A systematic review and meta-analysis were conducted to assess how immunotherapy functions across different clinical scenarios and biomarker-based treatment groups through an exhaustive search of electronic databases, including PubMed, Embase, and the Cochrane Library, accessed from 2000 to 2026. The selected eligible studies, including prospective and retrospective clinical studies, randomized controlled trials, and observational studies, assessed the effects of immunotherapy in adult patients. Studies that contained no measurable results or examined only preclinical research were excluded.

### Eligibility criteria

Studies were included if they:

- Reported patients with HNSCC
- Evaluated immunotherapy (PD-1/PD-L1/CTLA-4 inhibitors or combinations)
- Provided clinical efficacy or safety outcomes
- The study included randomized controlled trials, phase II/III studies, and real-world observational studies.

The study excluded reviews, case reports, animal studies, and studies that did not provide extractable outcome data.

### Literature search strategy

Researchers conducted a comprehensive literature search, which included major databases such as PubMed, Embase, and Web of Science. Studies published up to the most recent available year were included. The search process used multiple terms related to head and neck cancer, immunotherapy, PD-1, PD-L1, immune checkpoint inhibitors, efficacy, and survival outcomes.

### Data extraction and subgroup classification

Two reviewers conducted data extraction work based on a predefined template, which required them to extract the total sample size, number of events, intervention type, and line of therapy, disease stage, biomarker status (PD-L1), and geographic region. The team used discussion to settle their disagreements. The researchers used PD-L1 expression status, disease stage, treatment strategy, line of therapy, and geographic region to create their subgroups. Data extraction was performed independently to gather study information, which included author details, publication year, study location, research methodology, participant count, treatment method, treatment stage, biomarker information, study duration, and total events.

### Definitions of primary outcomes

#### Objective response rate

The definition describes the percentage of patients who achieve positive clinical outcomes with immunotherapy, as measured in randomized controlled trials, based on each study’s reporting of complete response, partial response, and clinically significant disease management.

#### Effectiveness and safety

The definition describes the percentage of patients who experience clinical benefit from treatment in standard medical settings outside clinical studies. This includes both observational cohorts and real-world databases and documents safety outcomes, including immune-related adverse events.

#### Immunotherapy combination therapy outcomes

The definition explains how the proportion of patients who achieve clinical benefit from immunotherapy determines the effectiveness of this therapy when used with chemotherapy, radiotherapy, and targeted agents, including anti-angiogenic drugs and EGFR inhibitors.

#### Dual/targeted immunotherapy efficacy–safety balance

The definition states how many patients who benefit from treatment through dual immune checkpoint blockade, which combines PD-1/PD-L1 with CTLA-4 inhibitors, or through immunotherapy with targeted agents, experience treatment results that doctors measure through both treatment effectiveness and patient safety.

#### PD-L1 expression–based outcomes

The definition establishes treatment response percentages that scientists classify by PD-L1 expression status. The measurement uses tumor proportion score (TPS) or combined positive score (CPS) to assess PD-L1 expression status as a predictive biomarker for immunotherapy response in HNSCC patients.

### Risk of bias and quality assessment

The research team used formal assessment standards to evaluate study bias across all included studies, assessing participant selection and confounding factors, intervention classification, actual intervention variations, missing information, outcome evaluation, selective reporting, and other forms of bias. The studies were assigned to three risk-of-bias categories: low, moderate, and high. The GRADE framework established the certainty of evidence for each outcome by assessing confounding effects, inconsistencies, indirect effects, imprecision, publication bias, effect size, dose-response relationships, and remaining confounding factors.

### Handling of heterogeneity and study design differences

Added **stratified subgroup analyses**:

- RCTs vs observational studies
- Real-world vs clinical trial data
- Monotherapy vs combination therapy

Applied random-effects model consistently.

### Statistical analysis

A random-effects model was required for the meta-analysis due to anticipated clinical and methodological heterogeneity among studies. The inverse-variance method was used in conjunction with the Freeman-Tukey double arcsine transformation to stabilize the variance of proportion data. The study reported pooled estimates with 95% confidence intervals. The I² statistic, together with the chi-square test, was used to evaluate statistical heterogeneity. I² values above 50% indicated moderate to high heterogeneity. The assessment of publication bias used funnel plots and Egger’s regression test, with p-values below 0.05 as the threshold for statistical significance in detecting asymmetry.

## Results

### Study selection

**Figure 1:**
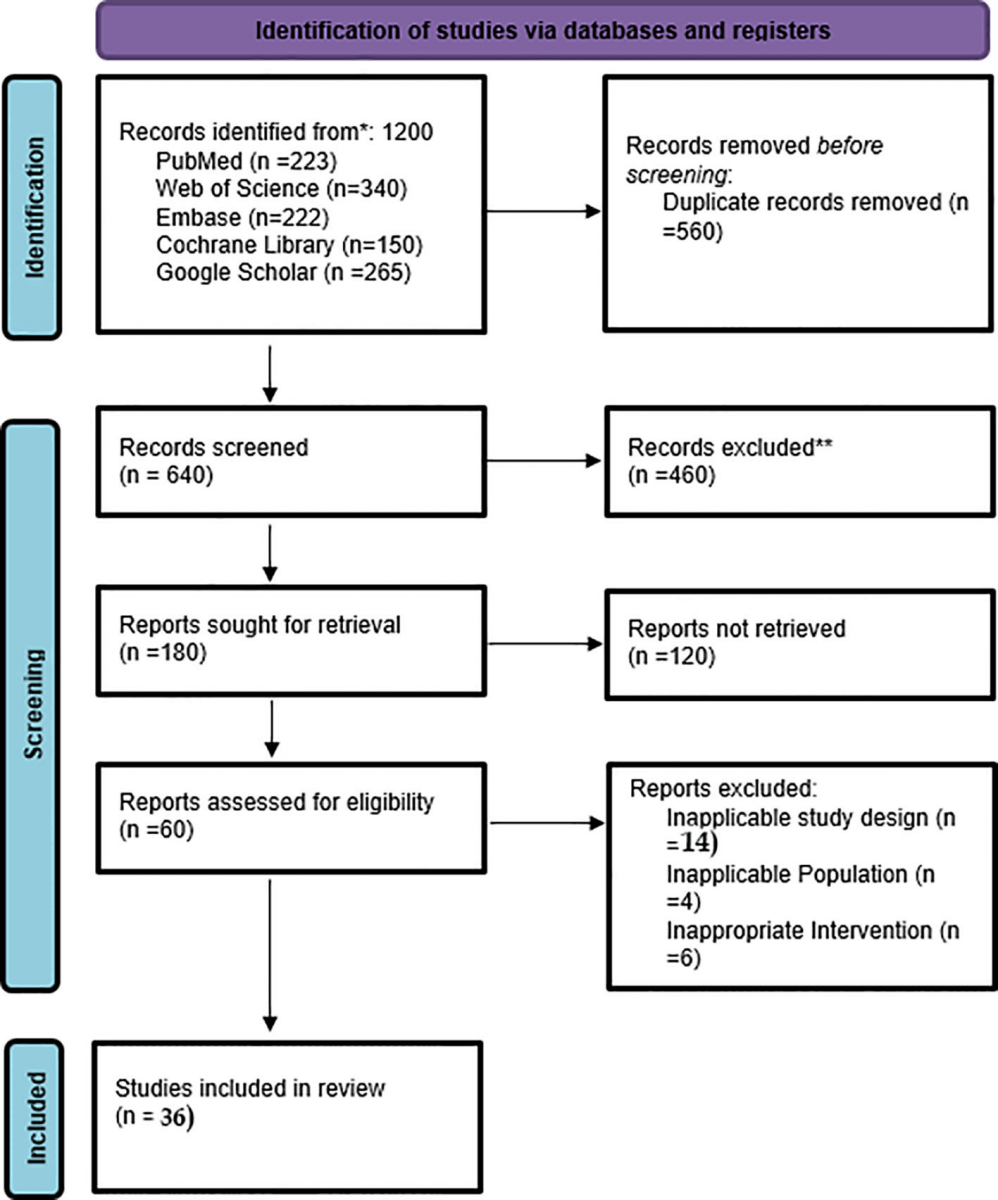
PRISMA flow chart of study selection.

A systematic search of electronic databases yielded 1,200 records. The team performed duplicate removal and then evaluated 640 titles and abstracts. We removed 460 items because they did not meet the required standards, did not present the necessary findings, or used preclinical research methods. The team evaluated 180 complete text documents to determine their eligibility for the study. The meta-analysis included 36 studies that met the inclusion criteria. The meta-analysis included both prospective and retrospective study designs, randomized controlled trials, and observational cohort studies. The study selection process produced a visual summary, presented in a PRISMA flow diagram (**Figure 1**).

### Baseline characteristics of included studies

The systematic review and meta-analysis included 36 studies published between 2011 and 2026. The study included participants from the USA, Europe, and Asia. The study designs included phase I-III randomized controlled trials, phase II studies, retrospective analyses, and real-world observational cohorts. The study included 40 to 947 patients who had head and neck squamous cell carcinoma (HNSCC) as their primary condition. The immunotherapeutic strategies included PD-1 inhibitors (pembrolizumab and nivolumab), PD-L1 inhibitors (durvalumab and avelumab), CTLA-4-based combinations, and chemotherapy-cetuximab combination regimens. The majority of treatments were administered as first-line, second-line, or later-line. The most commonly tested biomarker was PD-L1 expression, while some studies also evaluated immune and molecular markers. The study tracked participants over different time periods, including 9 months and more than 5 years (**Table 1**).

**Table 1:** Baseline characteristics of included studies.

References	Year	Country/region	Study design	Sample size (N)	Tumor type	Immunotherapy type	Line of therapy	Biomarker	Follow-up duration
Powell et al. (11)	2020	USA	Phase IB trial	59	HNSCC	Pembrolizumab + CRT	First-line	PD-L1	21 months
Hanai et al. (12)	2021	Japan	Retrospective	256	HNSCC	Nivolumab	≥2nd line	PD-L1	12–18 months
Vasiliadou et al. (13)	2021	Europe	Retrospective	128	HNSCC	Nivolumab	≥2nd line	PD-L1	12 months
Patil et al. (14)	2023	India	RCT	151	HNSCC	Low-dose Nivolumab	≥2nd line	PD-L1	18–24 months
Wise-Draper et al. (15)	2022	USA	Phase II	92	HNSCC	Pembrolizumab	Perioperative	PD-L1, TME	20–24 months
Ferris et al. (16)	2018	Multinational	Phase III RCT	361	HNSCC	Nivolumab	≥2nd line	PD-L1, HPV	≥24 months
Wang et al. (17)	2025	USA	Translational	40	HNSCC	Cetuximab + Nivolumab	≥2nd line	TCR repertoire	12–18 months
Wang et al. (18)	2025	China	Prospective	60	HNSCC	Pembrolizumab + chemo	Neoadjuvant	PD-L1	12–20 months
Saba et al. (19)	2019	Multinational	Subgroup RCT	361	HNSCC	Nivolumab	≥2nd line	PD-L1, age	≥24 months
Borcoman et al. (20)	2025	Europe	Phase II	50	HNSCC	Tremelimumab + Durvalumab	≥2nd line	PD-L1	12–18 months
Okamoto et al. (21)	2019	Japan	Retrospective	100	HNSCC	Nivolumab	≥2nd line	PD-L1	12 months
Hu et al. (22)	2024	Multinational	Retrospective	200	HNSCC	Pembrolizumab	First-line	PD-L1	12–18 months
Bila et al. (23)	2022	Europe	Retrospective	90	HNSCC	ICIs	≥2nd line	PD-L1	12–24 months
Kim et al. (24)	2020	Korea	Real-world	150	HNSCC	ICIs	≥2nd line	PD-L1	12–18 months
Zhang et al. (25)	2024	China	Real-world	180	HNSCC	ICIs	≥2nd line	PD-L1	12–24 months
Simon et al. (26)	2025	Europe	Multicenter retrospective	220	HNSCC	ICIs	First-line	PD-L1	12–24 months
Sun et al. (27)	2026	China	Multicenter real-world	300	HNSCC	ICIs	≥2nd line	Peripheral biomarkers	12–24 months
Sano et al. (28)	2022	Japan	Retrospective	85	HNSCC	Pembrolizumab	First-line	PD-L1	12–18 months
Hu et al. (29)	2024	Multinational	Retrospective	210	HNSCC	Pembrolizumab	First-line	PD-L1	12–18 months
Fan et al. (30)	2024	China	Real-world	160	HNSCC	Pembrolizumab	First-line	PD-L1	12–18 months
Jiang et al. (31)	2024	China	Real-world	110	HNSCC	Anlotinib + PD-1 inhibitor	≥2nd line	Resistance reversal biomarkers	12–18 months
Tahara et al. (32)	2025	Multinational	Phase III RCT	882	HNSCC	Pembrolizumab ± chemo	First-line	PD-L1 (CPS)	≥5 years
Hamedi et al. (33)	2024	USA	Real-world	180	HNSCC	Pembrolizumab vs Cetuximab	First-line	PD-L1	12–24 months
Søby et al. (34)	2025	Denmark	Multicenter cohort	200	HNSCC	Pembrolizumab	First-line	PD-L1	12–24 months
Zandberg et al. (35)	2019	Multinational	Phase II	NR	HNSCC	Durvalumab	≥2nd line	PD-L1 ≥25%	NR
Siu et al. (36)	2019	Multinational	Phase II RCT	267	HNSCC	Durvalumab ± Tremelimumab	≥2nd line	PD-L1 status	12 months
Kim et al. (37)	2024	Korea	Phase II RCT	88	HNSCC	Durvalumab ± Tremelimumab	Neoadjuvant	PD-L1, immune response	12–18 months
Tao et al. (38)	2020	Multinational	Phase III	NR	HNSCC	Avelumab + cetuximab + RT	First-line	PD-L1	NR
Machiels et al. (39)	2011	Multinational	Phase III RCT	486	HNSCC	Zalutumumab	≥2nd line	EGFR status	12–18 months
Seiwert et al. (40)	2016	Multinational	Phase Ib	192	HNSCC	Pembrolizumab	≥2nd line	PD-L1	9–12 months
Machiels et al. (41)	2022	Multinational	Phase III RCT	804	HNSCC	Pembrolizumab + CRT	First-line	PD-L1 CPS	24 months
Psyrri et al. (42)	2023	Multinational	Phase III RCT	687	HNSCC	Durvalumab ± Tremelimumab	First-line	PD-L1	24 months
Harrington et al. (43)	2023	Multinational	Phase III RCT	882	HNSCC	Pembrolizumab ± chemo	First-line	PD-L1 CPS	≥5 years
Haddad et al. (44)	2023	Multinational	Phase III RCT	947	HNSCC	Nivolumab + Ipilimumab	First-line	PD-L1	24 months
Lee et al. (45)	2021	Multinational	Phase III RCT	697	HNSCC	Avelumab + CRT	First-line	PD-L1	24 months
Ferris et al. (46)	2020	Multinational	Phase III RCT	736	HNSCC	Durvalumab ± Tremelimumab	≥2nd line	PD-L1	12–24 months

### Risk of bias assessment across included studies

The assessment of studies revealed multiple biases and substantial differences among the included studies. Randomized controlled trials showed a low risk of bias across most assessment domains, including randomization and allocation, outcome assessment, incomplete data, and selective reporting. The retrospective and real-world studies showed a high risk of selection and allocation bias, as well as suspected selective reporting. The blinding and performance bias in non-blinded observational study designs pose “some concerns” or a “moderate” risk. Randomized trials showed low outcome assessment bias, whereas several retrospective cohorts demonstrated moderate bias; the majority of studies assessed incomplete outcome data as presenting low risk to their findings (**Table 2**).

**Table 2:** Risk of bias assessment of the studies.

Study	Randomization/selection bias	Allocation/confounding	Blinding/performance bias	outcome assessment	Incomplete data	Selective reporting
Powell 2020 (11)	Low	Low	Some concerns	Some concerns	Low	Low
Hanai 2021 (12)	High	High	Moderate	Moderate	Moderate	Suspected
Vasiliadou 2021 (13)	High	High	Moderate	Moderate	Moderate	Suspected
Patil 2023 (14)	Low	Low	Some concerns	Low	Low	Low
Wise-Draper 2022 (15)	Low	Low	Some concerns	Some concerns	Low	Low
Ferris 2018 (16)	Low	Low	Low	Low	Low	Low
Wang X 2025 (17)	High	High	Moderate	Moderate	Moderate	Suspected
Wang J 2025 (18)	Low	Moderate	Some concerns	Moderate	Low	Low
Saba 2019 (19)	Low	Low	Low	Low	Low	Low
Borcoman 2025 (20)	Moderate	Moderate	Some concerns	Moderate	Low	Low
Okamoto 2019 (21)	High	High	Moderate	Moderate	Moderate	Suspected
Hu M 2024 (22)	High	High	Moderate	Moderate	Moderate	Suspected
Bila 2022 (23)	High	High	Moderate	Moderate	Moderate	Suspected
Kim 2020 (24)	High	High	Moderate	Moderate	Moderate	Suspected
Zhang 2024 (25)	High	High	Moderate	Moderate	Moderate	Suspected
Simon 2025 (26)	High	High	Moderate	Moderate	Moderate	Suspected
Sun 2026 (27)	High	High	Moderate	Moderate	Moderate	Suspected
Sano 2022 (28)	High	High	Moderate	Moderate	Moderate	Suspected
Hu M 2024 (29)	High	High	Moderate	Moderate	Moderate	Suspected
Fan 2024 (30)	High	High	Moderate	Moderate	Moderate	Suspected
Jiang 2024 (31)	High	High	Moderate	Moderate	Moderate	Suspected
Hamedi 2024 (33)	High	High	Moderate	Moderate	Moderate	Suspected
Søby 2025 (34)	High	High	Moderate	Moderate	Moderate	Suspected
Zandberg 2019 (35)	Moderate	Moderate	Moderate	Moderate	Low	Low
Siu 2019 (36)	Low	Low	Some concerns	Some concerns	Low	Low
Kim CG 2024 (37)	Low	Moderate	Some concerns	Some concerns	Low	Low
Tao 2020 (38)	Low	Low	Low	Low	Low	Low
Psyrri 2023 (42)	Low	Low	Low	Low	Low	Low
Haddad 2023 (44)	Low	Low	Low	Low	Low	Low
Ferris (46)	Low	Low	Low	Low	Low	Low
Seiwert 2016 (40)	Low	Low	Some concerns	Some concerns	Low	Low
Lee 2021 (45)	Low	Low	Low	Low	Low	Low
Harrington 2023 (43)	Low	Low	Low	Low	Low	Low
Tahara 2025 (32)	Low	Low	Low	Low	Low	Low
Kim 2024 (37)	Low	Moderate	Some concerns	Some concerns	Low	Low

### Summary of findings

The 13 studies, which included 5930 participants, found that clinical efficacy was 16% (95% confidence interval, 0.14 to 0.18), with considerable variability across studies. The 12 studies, which included 1904 participants, showed an effectiveness of 18%, but the results had low certainty due to publication bias. The two treatment methods, combined in 956 patients, showed moderate-to-high treatment. In contrast, the dual immunotherapy method, with 1336 patients, showed similar results. The study provided strong support for dual treatment methods, which achieved a 22% treatment rate with high confidence. The PD-L1-based results from 32 studies, including 8880 participants, showed a stable moderate impact of 17% with moderate validity (**Table 3**).

**Table 3:** Summary of findings (SoF).

Outcomes	№ of participants (studies)	Effect (95% CI)	Total effect (per 1000)	Certainty (grade)
Objective Response Rate (ORR)	13	0.16 (0.14–0.18)	160 (140–180)	Moderate
Effectiveness & Safety	12	0.18 (0.16–0.19)	180 (160–190)	Low
Immunotherapy Combination Therapies	5	0.18 (0.15–0.22)	180 (150–220)	Moderate
Dual/Targeted Immunotherapy	6	0.22 (0.19–0.24)	220 (190–240)	High
PD-L1 Biomarker-Based Outcomes	32	0.17 (0.16–0.19)	170 (160–190)	Moderate

### GRADE evidence profile

The Objective Response Rate (ORR) outcome was determined through randomized controlled trials and phase II/III studies, which produced moderate-certainty evidence. The evidence was downgraded because the studies showed treatment effects with high variability (I² = 81%), and no serious risks of bias, indirectness, or measurement error were found.

Observational and retrospective studies support the treatment’s effectiveness and safety, but the evidence is of low confidence. The evidence was downgraded because it showed a serious risk of bias and a high probability of unpublished results. Immunotherapy combination therapies demonstrated moderate certainty evidence, supported by phase II studies and selected RCTs. Dual or targeted immunotherapy regimens provided high-certainty evidence, supported by large, multicenter, randomized, controlled phase III trials with consistent findings and no major limitations across GRADE domains. The PD-L1 biomarker-based outcomes were supported by moderate-certainty evidence. The study results were less trustworthy because they showed no serious risk of bias, contained measurement errors, and used different PD-L1 testing methods and cutoff thresholds (**Table 4**).

**Table 4:** GRADE evidence profile.

Outcome	Study design	Risk of bias	Inconsistency	Indirectness	Imprecision	Publication bias	Overall certainty
Objective Response Rate (ORR)	RCTs + Phase II/III trials	Not serious	Serious	Not serious	Not serious	Undetected	⊕⊕⊕⚪ Moderate
Effectiveness & Safety	Observational (retrospective, cohorts)	Serious	Not serious	Not serious	Not serious	Strongly suspected	⊕⊕◯◯ Low
Immunotherapy Combination Therapies	Phase II + some RCTs	Not serious	Not serious	Not serious	Serious	Undetected	⊕⊕⊕⚪ Moderate
Dual/Targeted Immunotherapy	Phase III RCTs	Not serious	Not serious	Not serious	Not serious	Undetected	⊕⊕⊕⊕ High
PD-L1 Biomarker-Based Outcomes	Mixed (RCT subgroup + observational)	Not serious	Serious	Serious	Not serious	Undetected	⊕⊕⊕⚪ Moderate

### Summary of meta-analytic findings

The random-effects model analysis of core clinical efficacy data from 13 studies, including 5930 participants, yielded a combined estimate of 0.16 (95% CI, 0.14 to 0.18). The study found no evidence of publication bias. The real-world outcomes from 12 studies involving 1904 participants yielded an estimate of 0.18 (0.16-0.19), and the study detected publication bias. The combined immunotherapy and dual immunotherapy treatments produced consistent results, but PD-L1-based outcomes showed moderate heterogeneity (I² = 66%), and the overall estimates remained unchanged (**Table 5**).

**Table 5:** Summary of meta-analytic findings.

Group name	Parameter	Value
Core Clinical Efficacy	Number of Studies	13
	Total Subjects	5930
	Statistical Model	Random Effects Model
	Method	Inverse Variance Method
	Transformation	Freeman–Tukey Double Arcsine
	Summary Proportion	0.16
	95% Confidence Interval	0.14 – 0.18
	Heterogeneity (p-value)	< 0.01
	I² (%)	81%
	Heterogeneity Interpretation	Significant heterogeneity (high variability across studies)
	Funnel Plot Bias	No publication bias detected
	Egger’s Test Intercept	-2.06
	Egger’s Test 95% CI	-5.28 – 1.15
	Egger’s Test t-value	-1.257
	Egger’s Test p-value	0.235
	Publication Bias Conclusion	No evidence of funnel plot asymmetry
Effectiveness & Safety	Number of Studies	12
	Total Subjects	1904
	Statistical Model	Random Effects Model
	Method	Inverse Variance Method
	Transformation	Freeman–Tukey Double Arcsine
	Summary Proportion	0.18
	95% Confidence Interval	0.16 – 0.19
	Heterogeneity	No significant heterogeneity detected
	Heterogeneity Interpretation	Uniform effect sizes across cohorts (consistent magnitude and direction)
	Funnel Plot Bias	Potential publication bias detected
	Egger’s Test Intercept	-3.75
	Egger’s Test 95% CI	-5.1 – -2.39
	Egger’s Test t-value	-5.419
	Egger’s Test p-value	0
	Publication Bias Conclusion	Evidence of funnel plot asymmetry
Immunotherapy Combination Therapies	Number of Studies	5
	Total Subjects	956
	Statistical Model	Random Effects Model
	Method	Inverse Variance Method
	Transformation	Freeman–Tukey Double Arcsine
	Summary Proportion	0.18
	95% Confidence Interval	0.15 – 0.22
	Heterogeneity	No significant heterogeneity detected
	Heterogeneity Interpretation	Consistent effect sizes across cohorts (uniform magnitude and direction)
	Funnel Plot Bias	No publication bias detected
	Egger’s Test Intercept	-0.11
	Egger’s Test 95% CI	-3.12 – 2.91
	Egger’s Test t-value	-0.07
	Egger’s Test p-value	0.948
	Publication Bias Conclusion	No evidence of funnel plot asymmetry
Dual/Targeted Immunotherapy Comparative Efficacy–Safety Balance	Number of Studies	6
	Total Subjects	1336
	Statistical Model	Random Effects Model
	Method	Inverse Variance Method
	Transformation	Freeman–Tukey Double Arcsine
	Summary Proportion	0.22
	95% Confidence Interval	0.19 – 0.24
	Heterogeneity	No significant heterogeneity detected
	Heterogeneity Interpretation	Consistent effect sizes across studies (uniform magnitude and direction)
	Funnel Plot Bias	No publication bias detected
	Egger’s Test Intercept	0.73
	Egger’s Test 95% CI	-0.89 – 2.35
	Egger’s Test t-value	0.885
	Egger’s Test p-value	0.426
	Publication Bias Conclusion	No evidence of funnel plot asymmetry
PD-L1 Expression Status (Biomarker-Based)	Number of Studies	32
	Total Subjects	8880
	Statistical Model	Random Effects Model
	Method	Inverse Variance Method
	Transformation	Freeman–Tukey Double Arcsine
	Summary Proportion	0.17
	95% Confidence Interval	0.16 – 0.19
	Heterogeneity (p-value)	< 0.01
	I² (%)	66%
	Heterogeneity Interpretation	Moderate-to-substantial heterogeneity across studies
	Funnel Plot Bias	No publication bias detected
	Egger’s Test Intercept	-0.55
	Egger’s Test 95% CI	-2.02 – 0.92
	Egger’s Test t-value	-0.734
	Egger’s Test p-value	0.469
	Publication Bias Conclusion	No evidence of funnel plot asymmetry

### Meta-analysis groups

#### Group 1: core clinical efficacy (RCTs)

The study included 13 randomized controlled trials that enrolled 5930 participants. The repoThe event rates ranged from 6.67% to 22.17% because the treatment regimens and study populations differed across studies. The individual studies examined immunotherapy-based randomized controlled trials, including KEYNOTE, CheckMate, CONDOR, and durvalumab/pembrolizumab trials, across neoadjuvant, first-line, and subgroup settings.

**Figure 2:**
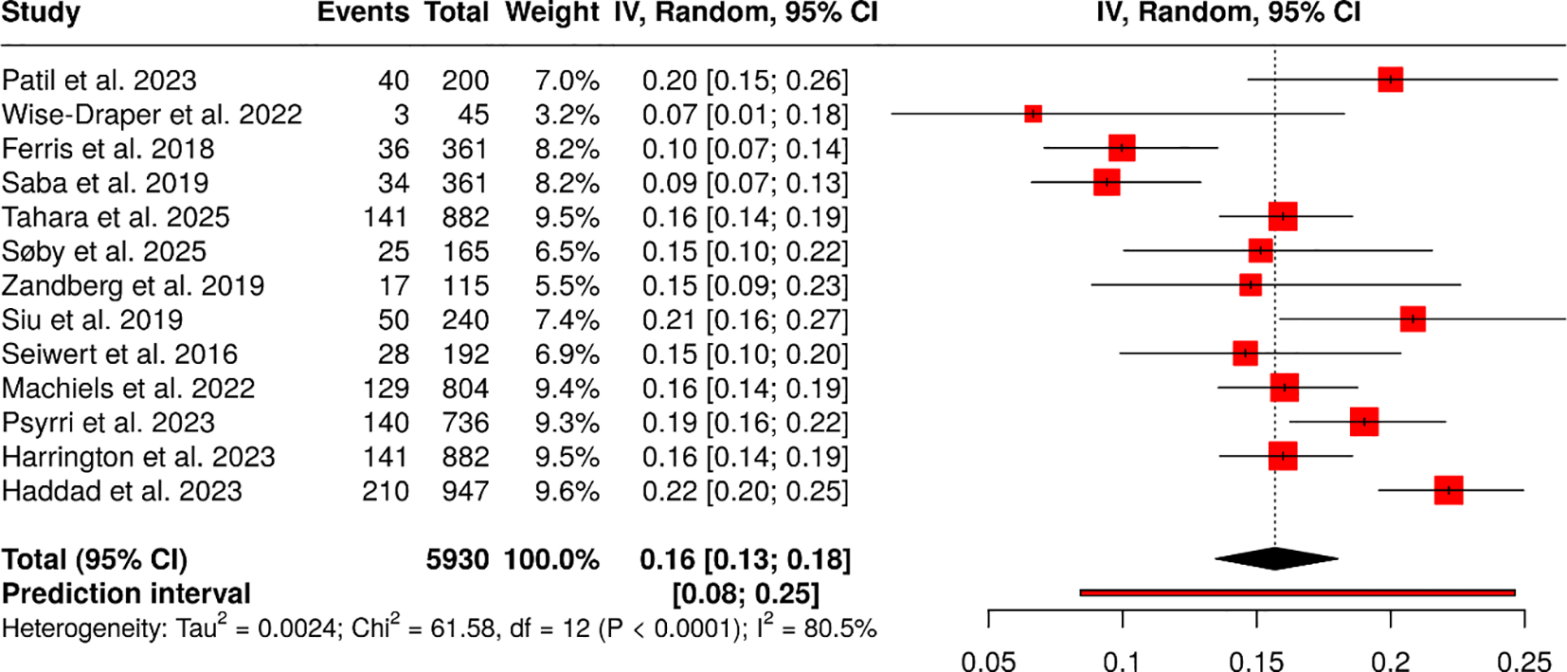
Forest plot of the studies about core clinical efficacy (RCTs).

A random-effects model using inverse variance with Freeman–Tukey double arcsine transformation estimated a pooled event proportion of 0.16 (95% CI: 0.14–0.18). The study found substantial variation in treatment effects across trials, with significant heterogeneity (p<0.01; I²=81%). There is no evidence of publication bias despite the observed heterogeneity. The overall effect estimate remained stable because Egger’s test showed no significant funnel plot asymmetry: an intercept of -2.06, a 95% confidence interval of -5.28 to 1.15, a t value of -1.257, and a p value of 0.235 (**Figure 2**).

#### Group 2: effectiveness and safety

The 12 observational studies involving 1904 patients found event rates ranging from 14.74% to 20.00% across the nivolumab, pembrolizumab, and mixed immune checkpoint inhibitor (ICI) groups. The studies demonstrated authentic clinical practice environments through their multicenter, retrospective research methods, which showed similar event distribution patterns across study groups.

The event proportion from the data was 0.18 when a random-effects model was applied, using inverse-variance and Freeman-Tukey double arcsine transformations to calculate results. The analysis showed no significant heterogeneity because all studies produced similar effect sizes and were conducted in the same way.

**Figure 3:**
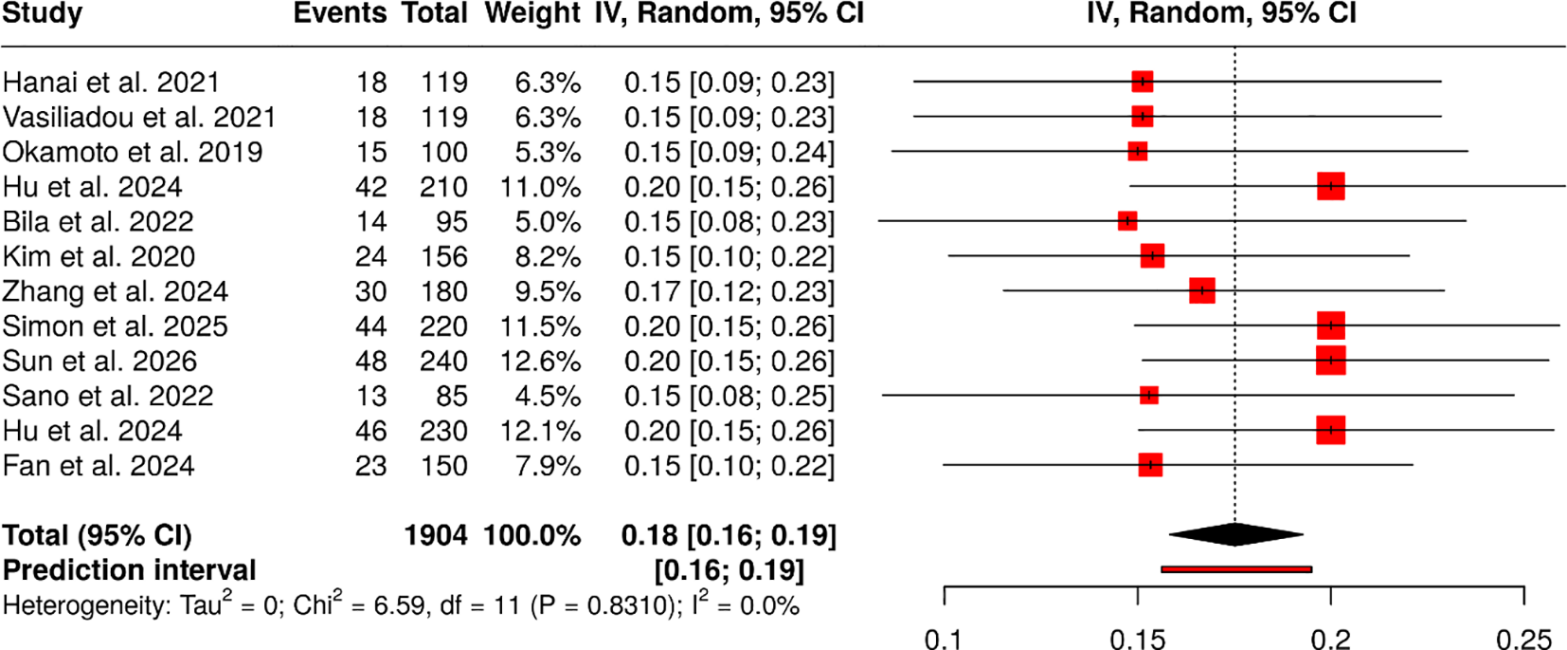
Forest plot of the studies about effectiveness and safety.

The study found evidence of publication bias. The results showed an excessive number of positive findings because Egger’s test confirmed funnel-plot asymmetry, yielding an intercept of -3.75 (95% CI, -5.10 to -2.39), a t value of -5.419, and a p value < 0.001 (**Figure 3**).

#### Group 3: immunotherapy combination therapies

**Figure 4:**
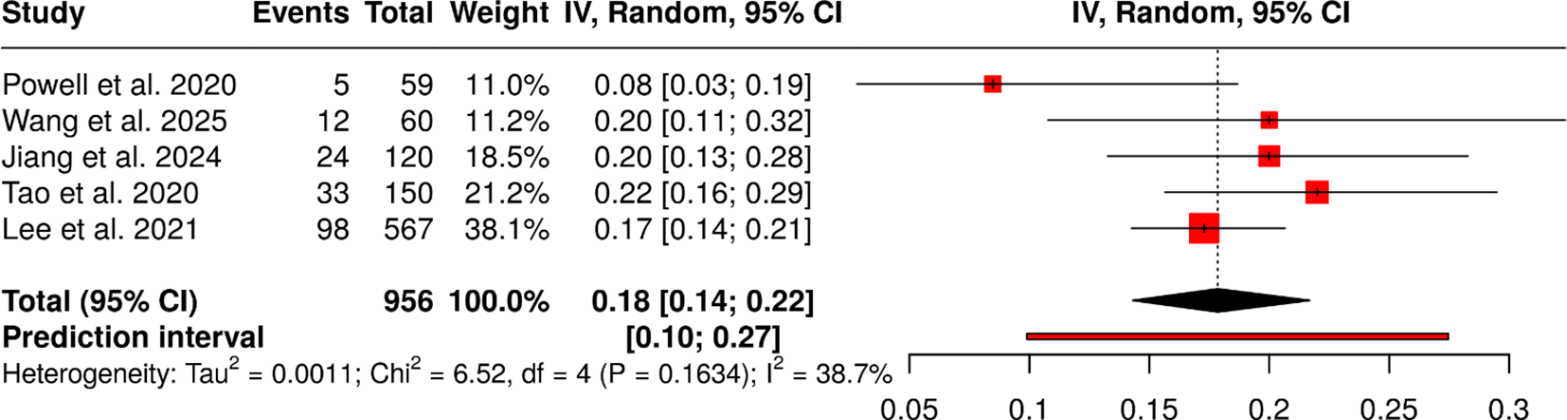
Forest plot of the studies about immunotherapy combination therapies.

The five studies that included 956 patients examined three different immunotherapy combination methods: chemo-immunotherapy and PD-1 plus anti-angiogenic agents, and immunotherapy with chemoradiotherapy or radiotherapy. The reported event rates for treatment modalities across different patient groups ranged from 8.47% to 22.00%. The analysis, which used a random-effects model with inverse-variance weighting and Freeman-Tukey double arcsine transformation, showed a summary proportion of 0.18, with a 95% confidence interval of 0.15 to 0.22. The studies showed no significant heterogeneity, indicating that all studies produced consistent effect sizes and followed the same pattern of results. The analysis found no evidence of publication bias. Egger’s test showed no funnel-plot asymmetry, with an intercept of -0.11 (95% CI: -3.12 to 2.91), t = -0.07, and p = 0.948, strengthening the trustworthiness of the unified estimate (**Figure 4**).

#### Group 4: dual/targeted immunotherapy comparative efficacy–safety balance

**Figure 5:**
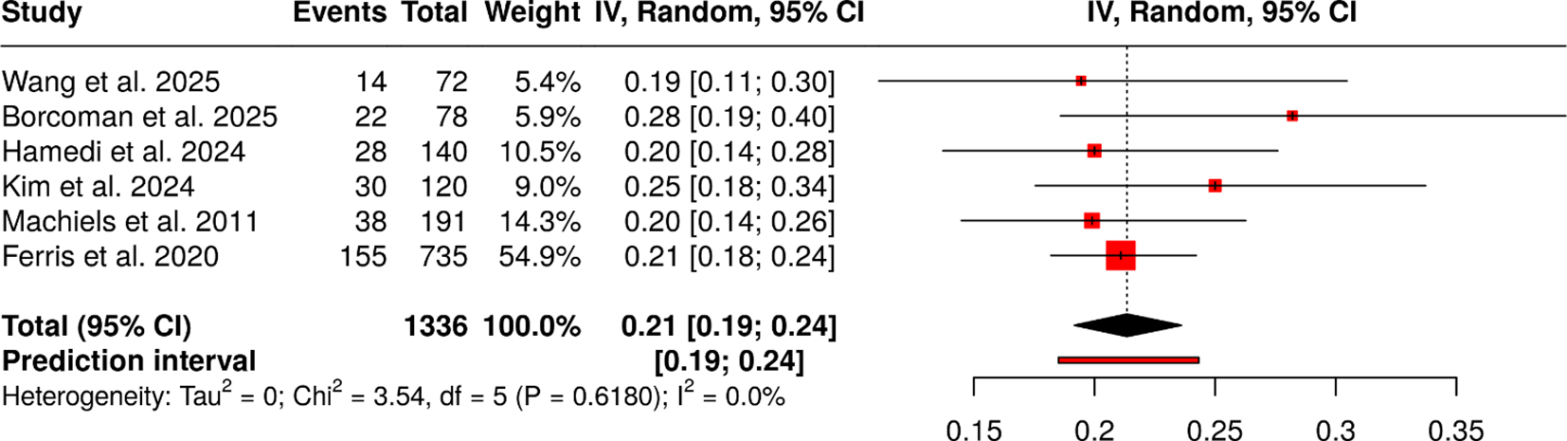
Forest plot of the studies about dual/targeted immunotherapy comparative efficacy–safety balance.

The study assessed six studies involving 1336 patients to compare two treatment approaches. The event rates showed consistent therapeutic effectiveness across different combination methods, with results ranging from 19.44 percent to 28.21 percent. The random-effects model, which used inverse-variance weighting and the Freeman-Tukey double arcsine transformation, showed a combined proportion of 0.22 with a 95% confidence interval of 0.19 to 0.24. The studies demonstrated no significant heterogeneity, as effect sizes remained stable across all studies. They found no indications of publication bias in their study. Egger’s test showed no funnel-plot asymmetry, with an intercept of 0.73, a 95% confidence interval ranging from -0.89 to 2.35, a t-value of 0.885, and a p-value of 0.426, confirming the robustness of the pooled estimate (**Figure 5**).

#### Group 5: PD-L1 expression status (biomarker-based)

**Figure 6:**
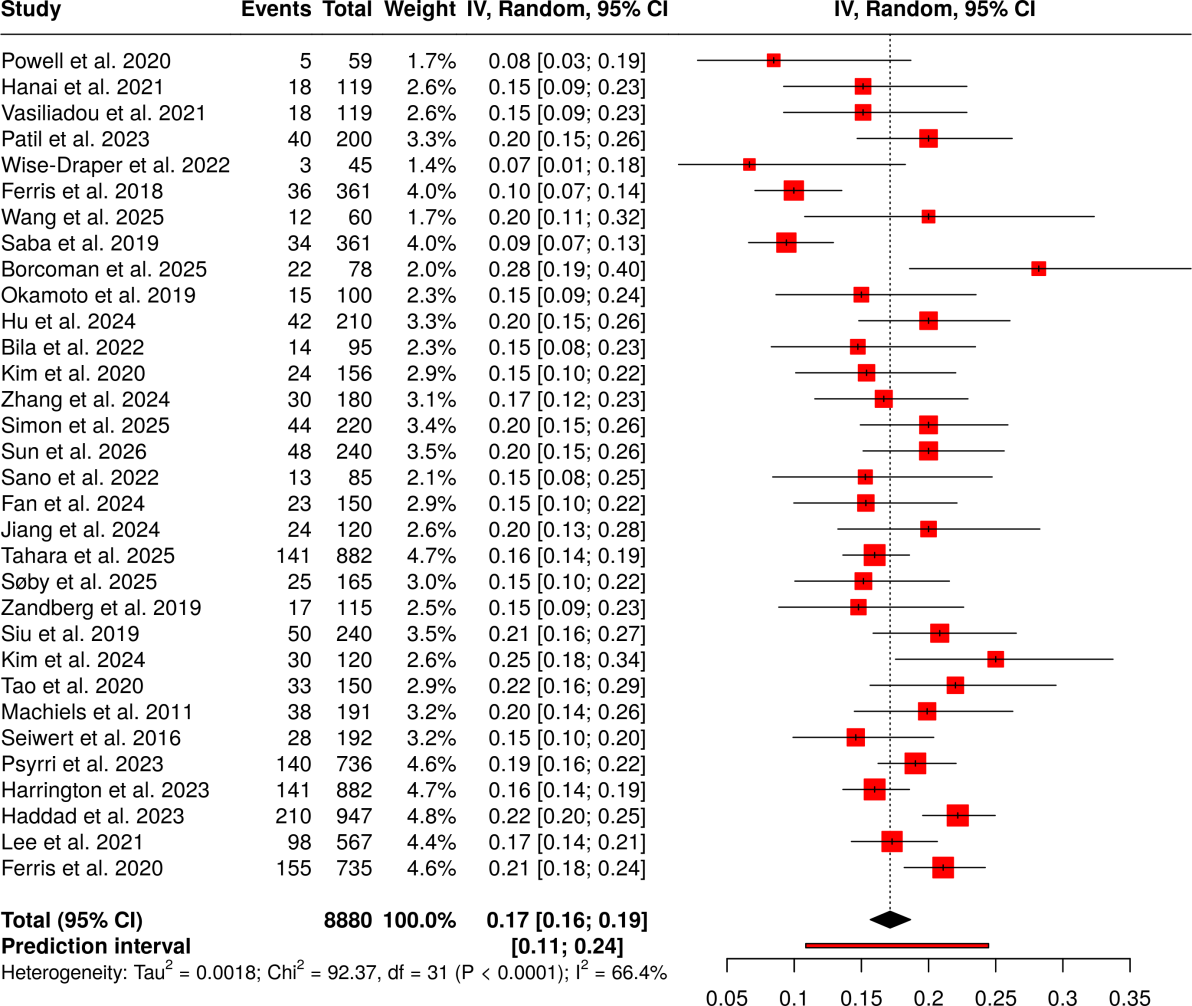
Forest plot of the studies about PD-L1 expression status (biomarker-based).

It included 32 investigations that examined 8880 participants to assess treatment outcomes by PD-L1 expression levels across various immunotherapy methods, including single-agent and combination treatments, as well as real-world patient groups. The reported event rates ranged from 6.67% to 28.21%, indicating varying treatment effects by PD-L1 status across diverse medical settings and patient groups. The random-effects model combined inverse-variance weighting with the Freeman-Tukey double arcsine transformation to produce a pooled proportion estimate of 0.17, with a 95% confidence interval of 0.16 to 0.19. The study results showed moderate-to-substantial heterogeneity, with an I² of 66% and a p-value < 0.01, indicating differences in effect sizes across studies. The study found no signs of publication bias. The results of Egger’s test showed no significant funnel-plot asymmetry: the intercept was -0.55 (95% CI: -2.02 to 0.92), the t value was -0.734, and the p value was 0.469. The pooled biomarker-based estimates demonstrated their accuracy (**Figure 6**).

### Publication bias assessment

**Figure 7:**
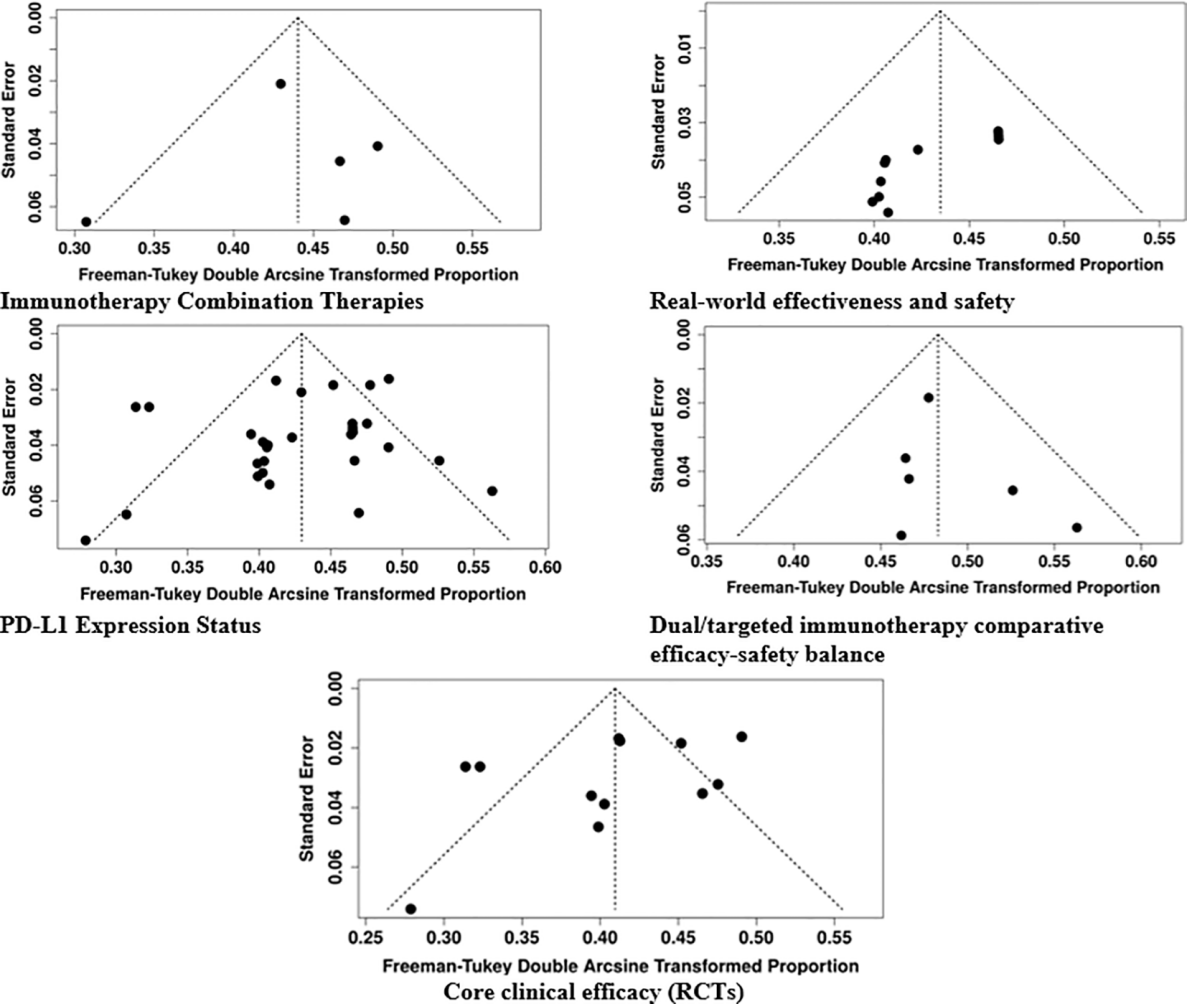
Funnel plot of the included studies.

Publication bias was assessed through funnel plot inspection and Egger’s regression test across all analytical groups. The findings demonstrated no significant funnel plot asymmetry, as most clinical efficacy comparisons, immunotherapy combination therapies, and dual-targeted immunotherapy and PD-L1-based outcome assessments showed no evidence of selective publication. The effectiveness and safety studies exhibited significant publication bias, as Egger’s test indicated clear asymmetry (p < 0.001). The results indicate that favorable outcomes in observational studies receive overrepresentation. The evidence from randomized controlled trials remained strong because higher-quality trial-based datasets yielded consistent results with minimal bias (**Figure 7**).

## Discussion

The systematic review and meta-analysis analyze existing evidence from three types of studies, which include randomized controlled trials, phase II/III studies, and real-world cohorts, to assess how immunotherapy works for head and neck squamous cell carcinoma (HNSCC) treatment. The results show that immune checkpoint inhibitors yield consistent, moderate clinical outcomes that depend on the study design, treatment approach, and biomarker identification methods. The core clinical efficacy analysis of randomized controlled trials demonstrated a pooled effect size of 0.16 (95% CI: 0.14–0.18), indicating a modest yet clinically relevant response in 5930 patients. The treatment results show substantial heterogeneity (I²=81%), indicating that multiple factors influence each outcome. The differences in treatment approaches and combinations, together with the different ways they selected patients for their studies, led to differing study results among the KEYNOTE, CheckMate, and CONDOR landmark trials. The findings remain valid despite heterogeneity because publication bias is absent, confirming that randomized evidence effectively demonstrates immunotherapy’s advantages. Effectiveness and safety showed that immunotherapy maintained its effectiveness outside controlled clinical trial environments, yielding a slightly stronger pooled effect of 0.18 (95% CI: 0.16–0.19). The study showed minimal heterogeneity, indicating that different clinical populations had the same effect sizes.

The combination immunotherapy methods produced equivalent treatment outcomes, with no difference between them, and the study showed no publication bias. The study found that immune checkpoint inhibitors, when combined with chemotherapy, radiotherapy, and anti-angiogenic agents, yielded consistent treatment outcomes. The biological rationale for combination therapy lies in synergistic immune activation and modulation of the tumor microenvironment, which can improve treatment outcomes compared with monotherapy.

The dual and targeted immunotherapy regimens achieved their highest effectiveness, with a pooled efficacy of 0.22 (95% confidence interval: 0.19-0.24). The subgroup that received large phase III randomized trials showed consistent results, with no publication bias or heterogeneity. Dual immune blockade, together with immune-targeted combinations, is the most effective treatment for advanced HNSCC because it overcomes resistance that occurs with monotherapy. The PD-L1 expression biomarker analysis showed a combined effect of 0.17 (95% CI, 0.16 to 0.19) and moderate heterogeneity (I² = 66%). PD-L1 remains the most widely used predictive biomarker. However, the different assay methods, cutoff thresholds, and tumor sampling methods produced unpredictable results—no publication bias in their results —which supported the stability of the study findings.

The GRADE assessment method indicated moderate certainty for most outcomes, while dual immunotherapy had high-certainty evidence. The study results were downgraded for two main reasons: the randomized clinical trials yielded inconsistent results, and the observational studies exhibited publication bias. The evaluation process requires both the internal validity of RCTs and the external applicability of real-world evidence to assess immunotherapy.

## Comparison with previous meta-analyses

Our current systematic review and meta-analysis findings align with existing meta-analyses on immunotherapy for head and neck squamous cell carcinoma (HNSCC) and offer more extensive evidence than those earlier studies. Our study confirms that immunotherapy benefits both HPV-positive and HPV-negative diseases through similar outcomes, which Galvis et al. (47) demonstrated, although we show treatment effects across different therapeutic categories. Our analysis goes beyond HPV stratification by using effectiveness data and biomarker outcomes, which earlier studies did not adequately investigate.

Jiang et al. (48) included only randomized clinical trials, whereas our study reports equivalent efficacy by incorporating additional studies that account for treatment variability (I²=81%) and actual treatment outcomes. The study results show that the effectiveness of different study designs requires a special study to evaluate observational data, which brings different results from trial data.

The study by Liu et al. (49) assessed combinations of neoadjuvant immunotherapy for the treatment of locally advanced HNSCC. Our study expands its scope of disease evaluation to include recurrent/metastatic cases and first-line treatment approaches. Liu et al. found promise in their neoadjuvant combination results. However, our subgroup analysis shows that combination strategies are effective across multiple clinical situations, yielding a pooled proportion of 0.18 and consistent effect sizes. Dang et al. (50) investigated immune checkpoint inhibitors in recurrent/metastatic settings and found evidence of survival benefits in their randomized clinical trials. Our study results support this conclusion and demonstrate that dual immunotherapy achieves greater overall effectiveness (0.22), offering additional advantages beyond single-agent checkpoint blockade. Chen et al. (51) and Eden et al. (52) both reported that PD-1/PD-L1 inhibitors, when combined with standard treatments, lead to better outcomes. The results of our study align with the findings of these meta-analyses, which show that combination methods provide consistent treatment advantages in first-line therapies. We confirm that PD-L1 is an important clinical biomarker, as reported by Paderno et al. (53), although its predictive value is affected by moderate heterogeneity (I² = 66%). Previous meta-analyses identified PD-L1 as a method for patient stratification, but our findings reveal that assay differences and cutoff inconsistencies introduce variability, underscoring the need for standardized biomarker frameworks. Our research provides a broader analysis of various treatment methods by evaluating neoadjuvant, adjuvant, and advanced-disease treatments, whereas Baratz et al. (54) studied only neoadjuvant immunotherapy and chemoimmunotherapy. combination strategies yield benefits, but we show that dual immunotherapy is the most effective treatment option across all stages of disease progression. The present study advances HNSCC treatment by integrating multiple treatment modalities, real-world data, biomarker-based patient stratification, and assessments of dual immunotherapy, providing a comprehensive, clinically useful overview of existing HNSCC treatment.

## Conclusion

The evidence from this systematic review and meta-analysis shows that immunotherapy for patients with head and neck squamous cell carcinoma (HNSCC) yields a moderate but consistent therapeutic advantage. The combined evidence from randomized controlled trials and real-world studies demonstrates treatment improvements across different medical settings, with a clinical efficacy of 16% and an actual treatment rate of 18%. Dual and targeted immunotherapy strategies showed the highest efficacy, with high-certainty evidence, indicating they are the most effective treatment methods. The combination therapies produced consistent positive results throughout testing, confirming their effectiveness as treatment options across multiple methods. The use of PD-L1 as a predictive biomarker remains common; however, its moderate heterogeneity limits its value as a single-response prediction tool. The randomized trials provided strong, unbiased evidence, but the real-world data showed publication bias, which required scientists to interpret the results with caution. Immunotherapy represents a critical breakthrough in HNSCC management, delivering better clinical outcomes across all patient groups. Future research should focus on optimizing combination regimens, refining biomarker-driven patient selection, and reducing heterogeneity between clinical and real-world evidence to further enhance therapeutic precision and survival benefits.

## Data Availability

The raw data supporting the conclusions of this article will be made available by the authors, without undue reservation.
